# The Mediating Role of Burnout and Secondary Traumatic Stress in the Relationship Between Perceived Stress and Quality of Life Among Nurses

**DOI:** 10.3390/ijerph23040540

**Published:** 2026-04-21

**Authors:** Marin Mamić, Tihomir Jovanović, Božica Lovrić, Gabriela Katharina Pomper, Ivana Mamić, Ivana Barać, Robert Lovrić, Goranka Rafaj, Danijela Kumpović, Ivan Vukoja

**Affiliations:** 1 General County Hospital Požega, Osječka 107, 34000 Požega, Croatia; bozicalovric@gmail.com (B.L.); gabrielakatharina.pomper@pozeska-bolnica.hr (G.K.P.); ivana.mamic@pozeska-bolnica.hr (I.M.); 2 Faculty of Dental Medicine and Health Osijek, Josip Juraj Strossmayer University of Osijek, Crkvena 21, 31000 Osijek, Croatia; tihomir.jovanovic@ozbpakrac-bhv.hr (T.J.); ibarac@fdmz.hr (I.B.); rlovric@fdmz.hr (R.L.); 3 General Hospital Pakrac and Hospital of Croatian Veterans, Bolnička 74, 34550 Pakrac, Croatia; 4 Faculty of Medicine, Josip Juraj Strossmayer University of Osijek, Josipa Huttlera 4, 31000 Osijek, Croatia; 5 Department of Nursing, University of Applied Sciences Ivanić-Grad, Moslavačka 13, 10310 Ivanić-Grad, Croatia; 6 Department of Nursing, University of Applied Sciences in Bjelovar, Trg Eugena Kvaternika 4, 43000 Bjelovar, Croatia; grafaj@vub.hr; 7 Sestre Milosrdnice University Hospital Center, Draškovićeva 19, 10000 Zagreb, Croatia; danijela.kumpovic@kbcsm.hr; 8 Faculty of Medicine, University of Rijeka, Ul. Braće Branchetta 20/1, 51000 Rijeka, Croatia

**Keywords:** nurses, perceived stress, burnout at work, quality of life, secondary traumatic stress, mediation

## Abstract

**Highlights:**

**Public health relevance—How does this work relate to a public health issue?**

- Occupational stress and burnout among nurses are important public health concerns because they affect the well-being of healthcare workers and may indirectly influence the quality and safety of patient care.
- Understanding how perceived stress is associated with nurses’ quality of life may help identify psychological mechanisms relevant to workforce sustainability in healthcare systems.

**Public health significance—Why is this work of significance to public health?**

- This study showed that perceived stress was associated with poorer physical and psychological quality of life among nurses.
- Burnout emerged as a significant indirect pathway in these associations, whereas secondary traumatic stress did not show a significant mediating role.

**Public health implications—What are the key implications or messages for practitioners, policy makers and/or researchers in public health?**

- Interventions aimed at improving nurses’ quality of life should focus not only on reducing stress, but also on the early recognition and prevention of burnout.
- Organizational measures such as improving working conditions, reducing excessive workload, and strengthening workplace support may contribute to better nurse well-being and indirectly to safer, higher-quality healthcare.

**Abstract:**

(1) Background: Nurses are exposed to occupational stressors that may impair their well-being and quality of life. This study examined whether burnout and secondary traumatic stress mediate the relationship between perceived stress and physical and psychological quality of life. (2) Methods: A cross-sectional study included 294 nurses employed at the Clinical Hospital Center Osijek, Croatia. Data were collected using the Perceived Stress Scale (PSS-10), the Burnout and Secondary Traumatic Stress subscales of the Professional Quality of Life Scale (ProQOL-5), and the physical and psychological domains of the WHOQOL-BREF. Pearson correlations and path analysis were used. (3) Results: Perceived stress showed significant negative effects on physical (β = −0.291; *p* < 0.001) and psychological quality of life (β = −0.217; *p* < 0.001), and positive effects on burnout (β = 0.230; *p* < 0.001) and secondary traumatic stress (β = 0.171; *p* = 0.002). Burnout significantly mediated both relationships, while secondary traumatic stress did not. The model explained 20.8% and 19.3% of variance in physical and psychological quality of life. (4) Conclusions: Burnout represents an important pathway linking perceived stress with poorer quality of life among nurses.

## 1. Introduction

Nurses’ quality of life is becoming an increasingly important topic of contemporary health research, especially due to the demanding and stressful nature of the nursing profession. High-quality and safe healthcare is one of the fundamental goals of health systems around the world, but the increase in work-related stress poses a serious threat to both health institutions and health workers [1,2,3,4]. Among health workers, nurses are particularly exposed to occupational stress due to their high workload, constant contact with patients, and the emotional demands of clinical practice [5,6,7]. The literature to date shows that stress can negatively affect the psychophysical state of an individual and can manifest itself through physical, emotional, psychological, and social difficulties [8,9,10]. Since these dimensions are also important components of quality of life, long-term exposure to occupational stress can have particularly adverse consequences for the general well-being of nurses [3,11]. In the nursing profession, these effects may be even more pronounced because the work of nurses involves close and continuous relationships with patients, often in extremely demanding and emotionally burdensome circumstances [8,12].

Nurses play a fundamental role in community health care, and in their daily work they are exposed to numerous stressors that can negatively affect their psychological and physical well-being and consequently impair their quality of life [3,4,13,14,15]. Nursing practice in public health institutions, such as hospitals, is particularly demanding because high work demands, patient pressure, rotating shift work schedules and unfavorable work environments can act as psychological risk factors [6,16,17]. Systematic reviews and meta-analyses show that health workers, and nurses in particular, are particularly vulnerable to various psychological difficulties, among which burnout and perceived stress stand out [13,18,19,20,21]. One of the most important consequences of long-term professional stress is burnout. Burnout is described as a psychological syndrome characterized by emotional exhaustion, depersonalization and a reduced sense of personal accomplishment in daily work [22,23]. Emotional exhaustion refers to a feeling of being overwhelmed and depleted of emotional and physical resources, while depersonalization involves a detached, negative, or cynical attitude toward patients. Reduced sense of personal accomplishment refers to a feeling of ineffectiveness and diminished professional competence [22]. Evidence among nursing staff suggests that burnout negatively impacts professional functioning and work capacity and may be directly related to low motivation, feelings of undervaluation, difficulty managing stress, psychological fatigue, and lower job satisfaction [16,17,24]. Consequently, burnout can significantly impair the general well-being and quality of life of nurses [3,11]. In addition, burnout is considered a serious work environment problem due to its negative consequences on employee productivity and overall functioning [25].

Perceived stress, on the other hand, is defined as a state of repeated tension caused by a situation that the individual assesses as threatening, while believing that he or she does not have enough resources to cope with such circumstances [13,26,27]. In the nursing profession, stress represents an important health risk factor because it can cause various physical, emotional and social difficulties that directly affect the quality of life of nurses, but also negatively affect the quality of care they provide to patients [3,4,24]. Previous findings also show that prolonged stress and burnout can negatively affect concentration, work engagement, productivity and a sense of achievement, thereby impairing not only professional functioning but also broader personal well-being [12,13,28]. The interaction of perceived stress and burnout can have a particularly adverse effect on the quality of life of nursing staff. Quality of life is viewed as the general well-being of an individual in the areas of physical, psychological, social, economic and environmental functioning [11,13,24]. Findings suggest that the simultaneous presence of elevated stress and burnout is closely associated with various psychological and physical difficulties that negatively affect the quality of life of nursing staff [3,29]. Therefore, it is reasonable to assume that burnout may represent an important mechanism through which perceived stress contributes to the deterioration of the quality of life of nurses. Given the importance of preserving the well-being and professional functioning of nurses, it is necessary to better understand the psychological factors that contribute to their quality of life. Examining the relationship between perceived stress, burnout and quality of life can contribute to a better understanding of the psychological processes that shape the well-being of nurses, but also help in the development of targeted interventions aimed at preserving their mental health, work ability and the quality of health care [30,31,32].

In addition to burnout, secondary traumatic stress is also an important construct for understanding the well-being of nurses [33]. It is a set of stress reactions and symptoms that arise from indirect exposure to traumatic experiences and suffering of others, which is particularly relevant in professions characterized by continuous and intensive contact with patients [34]. In everyday nursing practice, repeated exposure to pain, clinical deterioration, and death of patients can increase susceptibility to secondary traumatic stress, especially in conditions of increased perceived stress [35,36]. Previous research has shown that secondary traumatic stress is associated with less favorable psychological outcomes and poorer quality of life in healthcare workers, although such findings are more often described in emergency and highly demanding clinical settings and among newly employed nurses [35,36,37]. Accordingly, secondary traumatic stress can be viewed as a more specific mechanism through which perceived stress is associated with quality of life, alongside the broader and more general role of burnout in explaining nurses’ well-being.

These relationships may also be understood within the Job Demands–Resources (JD-R) model, according to which high job demands and insufficient resources contribute to stress and burnout, which may in turn impair employees’ well-being and quality of life [38,39]. In the nursing profession, this framework is particularly relevant because nurses are frequently exposed to heavy workload, emotional demands, and shift work, all of which may undermine their physical and psychological functioning [40].

Therefore, the aim of this study was to examine the mediating roles of burnout and secondary traumatic stress in the relationship between perceived stress and physical and psychological quality of life among nurses. Based on the existing literature and theoretical framework, the following hypotheses were formulated:

**H1.**  *Higher perceived stress will be associated with lower physical and psychological quality of life among nurses.*

**H2.**  *Higher perceived stress will be associated with higher levels of burnout and secondary traumatic stress.*

**H3.**  *Burnout will mediate the relationship between perceived stress and physical and psychological quality of life.*

**H4.**  *Secondary traumatic stress will mediate the relationship between perceived stress and physical and psychological quality of life.*

## 2. Materials and Methods

A cross-sectional study was conducted at the Osijek Hospital Center in the period from January to February 2026. The study included 294 nurses, of whom 247 were women (84%) and 47 were men (16%), and most of them had a secondary education, 131 (44.6%). The mean age of the respondents was M = 39.29 years (SD = 8.04). The respondents were included in the study by a convenience sample. The study was conducted in a single tertiary hospital setting to provide access to a clearly defined population of nurses working in comparable organizational and clinical settings. A convenience sample was used for the practical reasons of collecting data in a real hospital setting, where inclusion of respondents depends on their availability and voluntary participation during working hours. Such an approach is often used in cross-sectional studies examining occupational stress, burnout, and quality of life in nurses [3,4]. The study included nurses and medical technicians employed at the Clinical Hospital Center Osijek who were actively present at work at the time of the study and who voluntarily agreed to participate in the study. Data were collected using a paper questionnaire, which the respondents completed independently. It took approximately 10 to 15 min to complete the questionnaire.

Inclusion criteria were employment as a nurse or medical technician at the Clinical Hospital Center Osijek, age of 18 years or older, active presence at work during the study period, voluntary consent to participate, and the ability to understand and independently complete the questionnaire in Croatian. Employees who were not actively present at work at the time of the survey, for example due to sick leave, maternity or parental leave, annual leave, or other prolonged absence, were not included in the study. Questionnaires with incomplete responses or missing data on key study variables were excluded from the analysis.

### 2.1. Research Instruments

The survey used a questionnaire consisting of four parts. The first section was related to sociodemographic data, the second included the Perceived Stress Scale (PSS-10), the third included the Burnout and Secondary Traumatic Stress subscales from the Professional Quality of Life Scale, version 5 (ProQOL-5), and the fourth included the physical and psychological domains of the WHOQOL-BREF questionnaire.

The sociodemographic part of the questionnaire included data on gender, age, marital status, level of education, years of work experience, work environment, and shift work.

The Perceived Stress Scale (PSS-10) consists of 10 statements that assess the extent to which respondents have experienced their lives as unpredictable, difficult to control, and burdensome over the past month [41]. Responses are recorded on a scale from 0 (“Never”) to 4 (“Very Often”). Recent psychometric evidence has also supported the validity and reliability of the PSS-10 in nursing populations [42]. In addition, the Croatian-language version used in this study was based on a previously validated Croatian version of the PSS-10, with available studies supporting its reliability and validity in Croatian populations [43]. The total score is obtained by adding up the responses to all items, with a higher score indicating a higher level of perceived stress. In this study, the internal consistency of the questionnaire was very high (ω = 0.94).

The Professional Quality of Life Scale, version 5 (ProQOL-5), was used to assess professional quality of life [44]. Two subscales were used in this study: Burnout and Secondary Traumatic Stress. The Burnout subscale refers to emotional exhaustion, feelings of professional burden, and reduced work efficiency, while the Secondary Traumatic Stress subscale includes symptoms associated with indirect exposure to other people’s suffering and traumatic experiences. Each of these subscales contains 10 items, and responses are given on a scale from 1 (“Never”) to 5 (“Very often”). The score for each subscale is ex-pressed as the sum of responses, with a higher score indicating a greater severity of the observed phenomenon. The ProQOL-5 has also been examined in nursing populations, including Slovenian and Croatian nurses, with findings supporting its use while also indicating some psychometric limitations of the burnout subscale [45]. Accordingly, the Croatian-language use of the ProQOL-5 in this study was supported by evidence from a validation study conducted among Slovenian and Croatian nurses [46]. Both subscales in this study showed very high internal consistency (Burnout: ω = 0.94; Secondary Traumatic Stress: ω = 0.94).

The WHOQOL-BREF [47] was used to assess quality of life. The instrument includes 26 items divided into four domains. In this study, only the physical and psychological domains were analyzed because they were considered the most conceptually proximal outcomes in relation to perceived stress, burnout, and secondary traumatic stress, whereas the social and environmental domains were beyond the primary scope of the present model. Responses are given on a scale from 1 to 5, with higher values indicating a more favorable assessment of quality of life. In addition to the original WHO reference, subsequent psychometric studies have confirmed the suitability of the WHOQOL-BREF for assessing quality of life across diverse populations [47]. The Croatian version used in this study was based on a previously validated Croatian version of the WHOQOL-BREF, with available studies supporting its reliability and validity in Croatian populations [48]. In this study, the questionnaire showed satisfactory to very high reliability, with McDonald’s omega being 0.90 for the physical domain and 0.89 for the psychological domain.

### 2.2. Ethical Considerations

The study was conducted in accordance with the principles of the Declaration of Helsinki and was approved by the Ethics Committee of the University Hospital Centre Osijek, Croatia (protocol code: R1-14052-10/2025; approved on 13 December 2025). Participation in the study was voluntary and anonymous. Before completing the questionnaire, all participants were informed about the purpose of the study, the confidentiality of the collected data, and their right to withdraw from participation at any time without consequences. Written informed consent was obtained from all participants prior to data collection.

### 2.3. Statistical Methods

Descriptive statistical methods were used to present the basic characteristics of the sample and the research variables. Categorical variables were presented as absolute and relative frequencies, and numerical variables as arithmetic mean and standard deviation. The normality of the distribution of numerical variables was tested using the Kolmogorov–Smirnov test. Although statistically significant deviations from normal distribution were found for some variables, indicators of asymmetry and flattening indicated an approximately normal distribution, so considering the sample size, the application of parametric procedures was assessed as appropriate.

The mutual relationships between perceived stress, burnout at work, secondary traumatic stress, and physical and psychological dimensions of quality of life were examined using the Pearson correlation coefficient. The reliability of the applied measuring instruments was assessed using the McDonald omega coefficient.

Before implementing structural equation modeling, the basic assumptions of the regression analysis were checked. Multicollinearity was assessed using tolerance and variance inflation factors. Tolerance values ranged from 0.927 to 0.961, while VIF values ranged from 1.041 to 1.078, indicating the absence of multicollinearity problems. The independence of the residuals was assessed using the Durbin–Watson indicator. The value obtained for the model with the physical dimension of quality of life was 1.923, and for the model with the psychological dimension was 1.866, indicating that the assumption of independence of errors was met.

Structural equation modeling was used to examine the direct and indirect relationships between perceived stress, burnout at work, secondary traumatic stress, and the physical and psychological dimensions of quality of life. In this model, perceived stress served as a predictor variable, burnout at work and secondary traumatic stress as mediator variables, while the physical and psychological dimensions of quality of life were outcome variables. Model parameters were estimated using a robust maximum likelihood estimator (MLR), with robust standard errors and 95% confidence intervals. Direct, indirect and total effects were estimated within the mediation model. All analyses were performed in the statistical package JASP, version 0.96.0 [49]. The level of statistical significance was set at *p* < 0.05.

## 3. Results

The basic fit indices of the structural model are presented. Since this is a saturated model (df = 0), global fit indices cannot be interpreted in the usual way. As expected, the marginal values of fit indices were obtained, including χ^2^ < 0.001, CFI = 1.00, TLI = 1.00, NFI = 1.00, IFI = 1.00, RNI = 1.00 and SRMR < 0.001, reflecting the fact that the saturated model perfectly reproduces the observed relationships among the variables. Therefore, the assessment of the model in this research is based primarily on the interpretation of standardized regression coefficients, indirect effects and explained variance of the outcome, and not on global indicators of model fit. The information criteria were AIC = 3201, BIC = 3253 and SSABIC = 3208 (Table 1).

Table 2 presents descriptive indicators for physical and psychological quality of life, perceived stress, burnout at work, and secondary traumatic stress. Respondents scored relatively higher on average on the quality of life dimensions, while the average values for perceived stress and professional burden were moderate (Table 2).

Table 3 shows the Pearson correlations between physical and psychological quality of life, perceived stress, burnout at work and secondary traumatic stress. A low but statistically significant positive correlation was found between physical and psychological quality of life (r = 0.160, *p* = 0.006). Perceived stress was statistically significantly negatively associated with both dimensions of quality of life, namely physical quality of life (r = −0.362, *p* < 0.001) and psychological quality of life (r = −0.304, *p* < 0.001). At the same time, perceived stress was positively associated with burnout at work (r = 0.230, *p* < 0.001) and secondary traumatic stress (r = 0.171, *p* = 0.003). Burnout at work showed a statistically significant negative association with both physical quality of life (r = −0.351, *p* < 0.001) and psychological quality of life (r = −0.359, *p* < 0.001). Similarly, secondary traumatic stress was statistically significantly negatively associated with physical quality of life (r = −0.129, *p* = 0.027) and psychological quality of life (r = −0.188, *p* = 0.001). Overall, the associations with quality of life were stronger for burnout at work than for secondary traumatic stress (Table 3).

Table 4 shows the explained variance of mediators and outcomes in the structural model. The results showed that the model explained 5.3% of the variance in burnout at work and 2.9% of the variance in secondary traumatic stress. At the same time, the model explained 20.8% of the variance in physical quality of life and 19.3% of the variance in psychological quality of life, indicating a greater explanatory power of the model for outcome variables than for mediators (Table 4).

Table 5 shows the standardized regression coefficients in the structural model. The results showed that perceived stress had a statistically significant positive effect on burnout at work (β = 0.230, *p* < 0.001) and secondary traumatic stress (β = 0.171, *p* = 0.002). At the same time, perceived stress had a statistically significant negative direct effect on physical quality of life (β = −0.291, *p* < 0.001) and psychological quality of life (β = −0.217, *p* < 0.001). Burnout at work was also a significant negative predictor of physical quality of life (β = −0.278, *p* < 0.001) and psychological quality of life (β = −0.294, *p* < 0.001). Secondary traumatic stress was not a significant predictor of physical quality of life (β = −0.041, *p* = 0.463), but was a significant negative predictor of psychological quality of life (β = −0.111, *p* = 0.036) (Table 5).

Table 6 shows the indirect and total effects in the mediation model. The results showed that burnout at work is a significant mediator in the relationship between perceived stress and physical quality of life (β = −0.064, *p* < 0.001) and between perceived stress and psychological quality of life (β = −0.068, *p* < 0.001). On the other hand, the mediating role of secondary traumatic stress was not confirmed either for physical quality of life (β = −0.007, *p* = 0.482) or for psychological quality of life (β = −0.019, *p* = 0.072). The total indirect effect of perceived stress was statistically significant for both physical quality of life (β = −0.071, *p* = 0.001) and psychological quality of life (β = −0.087, *p* < 0.001). Also, the overall effect of perceived stress remained statistically significant for physical quality of life (β = −0.362, *p* < 0.001) and psychological quality of life (β = −0.304, *p* < 0.001), indicating partial mediation (Table 6).

## 4. Discussion

The aim of this study was to examine the mediating roles of burnout and secondary traumatic stress in the relationship between perceived stress and physical and psychological dimensions of quality of life among nurses. The results showed that perceived stress had a statistically significant negative direct effect on the physical and psychological dimensions of quality of life, while it had a significant positive effect on burnout and secondary traumatic stress. Burnout was also found to be a significant mediator in the relationship between perceived stress and both examined dimensions of quality of life, while the mediating role of secondary traumatic stress was not confirmed. However, given the cross-sectional design of the study, these findings should be interpreted as statistical and associative rather than causal.

This pattern of findings is largely consistent with previous research, which shows that higher levels of occupational stress among nurses are associated with poorer quality of life and poorer general well-being [3,4]. For example, Babapour et al. found that occupational stress among nurses is negatively associated with quality of life and caring behaviors [3], while Sarafis et al. reported that occupational stress was negatively associated with health-related quality of life in nurses [4]. The finding of a positive effect of perceived stress on burnout at work is also consistent with previous findings that long-term exposure to professional demands and psychosocial burdens favors the development of burnout among hospital workers and nurses [6,23]. These findings are also consistent with broader evidence showing that occupational stress among nurses is linked not only to reduced well-being, but also to poorer work functioning, lower job satisfaction, and reduced health-related quality of life [3,4,13,14].

Possible reasons for such results lie in the very nature of the nursing profession, which includes high workload, shift and night work, constant responsibility for patients, emotionally demanding clinical situations, and continuous exposure to suffering, pain, and uncertainty [6,12]. In such circumstances, nurses may experience their own professional demands as excessive and difficult to control, which increases the level of perceived stress. Long-term exposure to such stressors can lead to the depletion of emotional and physical resources, the development of a feeling of professional overload, and a decrease in personal well-being, which is consequently reflected in a lower quality of life [3,11].

Of particular importance is the finding that burnout was identified as a significant mediator in the relationship between perceived stress and physical and psychological quality of life. This finding is largely consistent with previous research, although the number of studies that have directly examined burnout as a statistical mediator in the association between perceived stress and quality of life among nurses is still limited. Previous research consistently shows that stress and emotional exhaustion are associated with poorer work and health-related quality of life in nurses, which supports the interpretation of burnout as an important factor associated with nurses’ well-being and functioning in the context of occupational stress [37,38]. In this sense, the obtained result that burnout mediates the relationship between perceived stress and physical and psychological dimensions of quality of life can be considered a logical extension of existing knowledge. In other words, the findings suggest that perceived stress was associated with quality of life both directly and indirectly within the tested model, with burnout accounting for part of this association. Nurses with higher levels of perceived stress also tended to report more burnout symptoms, and higher burnout was associated with poorer physical and psychological functioning. A possible explanation is that the repeated experience of professional demands as excessive and difficult to control may gradually deplete nurses’ emotional and physical resources, reduce their sense of professional efficacy, and be associated with poorer physical and psychological well-being. This finding is in line with the theoretical premise that burnout is one of the key consequences of chronic professional stress and that it is closely related to lower job satisfaction, lower professional effectiveness and poorer general well-being [22,25]. These interpretations should nevertheless be considered in light of the cross-sectional nature of the data.

The finding that burnout at work was a significant predictor of both physical and psychological quality of life is particularly important in the context of the nursing profession. Similar associations between burnout-related dimensions of professional quality of life and adverse occupational outcomes in nurses have also been reported in previous research [50]. Although burnout is rooted in the occupational context, its consequences are not limited to work-related functioning, but may extend to broader physical, emotional, and psychological well-being. Burnout at work does not only reflect emotional exhaustion, but can also be associated with physical fatigue, reduced energy, poorer recovery, greater feelings of burden, and a generally less favorable assessment of one’s own functioning. This may help explain why burnout was associated not only with the psychological, but also with the physical dimension of quality of life. The results therefore suggest that, in order to preserve nurses’ quality of life, it may be important to focus not only on reducing current stress, but also on preventing burnout as a longer-term consequence of professional burden [51,52,53].

When it comes to secondary traumatic stress, the results showed that perceived stress had a positive effect on this variable as well, which is also in line with recent research indicating that higher professional, or nursing, stress is associated with higher secondary traumatic stress, although the number of studies that directly examine the relationship between perceived stress and secondary traumatic stress among nurses is still limited [54,55]. A possible reason for such a finding is the fact that nurses are exposed not only to general organizational and work demands in their daily work, but also to intense emotional experiences of patients, their families, and situations of illness, suffering, deterioration, and death. In circumstances of increased stress, such exposure may further increase the emotional sensitivity and vulnerability of healthcare workers to indirect traumatic reactions. However, although secondary traumatic stress was a significant predictor of the psychological, but not the physical, dimension of quality of life, its mediating role was not confirmed in this study. Such a finding is not entirely unexpected. Previous research suggests that secondary traumatic stress may not have the same strong and stable mediating role as burnout; for example, in a study of newly graduated nurses, no significant mediating effect of secondary traumatic stress was found, whereas burnout showed a more important mediating role in explaining adverse outcomes [55]. This may also be explained by the fact that secondary traumatic stress has been more consistently reported in specific clinical contexts, such as emergency settings, where exposure to acute patient suffering and traumatic events is more frequent [54,55]. In contrast, the present study included a broader hospital-based sample, which may partly explain the weaker and non-significant mediating role of secondary traumatic stress. One possible explanation is that secondary traumatic stress reflects a more specific form of professional strain associated with indirect exposure to the suffering and trauma of others, whereas burnout is a broader and more general indicator of long-term professional exhaustion. Therefore, burnout may better capture the cumulative daily burden of nursing work and its association with general quality of life [34,37]. Therefore, it is possible that burnout better captures the daily cumulative workload of nurses and its effect on general quality of life, while secondary traumatic stress has a narrower and more selective contribution, especially in the psychological domain. The results obtained further suggest partial, rather than complete, mediation, since the direct effects of perceived stress on both dimensions of quality of life remained statistically significant even after the inclusion of mediators. This suggests that burnout accounted for an important part of the association between perceived stress and quality of life, but not the entire association. In other words, within the tested model, perceived stress was associated with quality of life both directly and indirectly through burnout. In addition, the model explained 20.8% of the variance in the physical and 19.3% of the variance in the psychological dimensions of quality of life, indicating moderate explanatory power of the model. This finding suggests that perceived stress, burnout, and secondary traumatic stress are important, but not the only, factors associated with nurses’ quality of life. Other individual, professional, and organizational factors, such as social support, work environment, work experience, coping strategies, job satisfaction, and general health, probably also influence quality of life. However, the fact that the examined model explained approximately one-fifth of the variance in both dimensions of quality of life confirms that stress and professional exhaustion have a clinically and practically relevant role in understanding nurses’ well-being. In addition, the exclusion of the social and environmental domains of quality of life may have narrowed the scope of the present findings, given that these domains may also be relevant to workplace support and organizational conditions.

Therefore, the findings suggest that burnout may represent an important pathway linking perceived stress with poorer quality of life among nurses, whereas the role of secondary traumatic stress appears more specific and less consistent. In practical terms, these findings suggest that interventions aimed at preserving nurses’ quality of life should focus not only on reducing perceived stress, but also on the early recognition and prevention of professional burnout. Such an approach could contribute not only to better well-being of nurses, but also to the quality and safety of healthcare.

Practical implications

The results obtained may also have important practical implications. Since burnout has been identified as a key mechanism linking perceived stress and quality of life, preventive and intervention activities in the work environment should focus on early recognition of burnout symptoms, strengthening stress coping strategies and improving organizational working conditions. This includes ensuring adequate support for employees, reducing excessive workload, better organizing shift work, encouraging team support and strengthening employees’ psychological resources. Ultimately, investing in nurses’ well-being not only contributes to their health and quality of life, but can also have a positive impact on patient safety and the quality of healthcare provided.

The present findings may also have theoretical implications. They suggest that burnout may represent a broader and more stable pathway linking perceived stress with physical and psychological quality of life, whereas secondary traumatic stress may reflect a more context-specific form of occupational strain associated with indirect exposure to patients’ suffering. This distinction may help explain why burnout showed a clearer mediating role in the present study. Future research should examine the role of secondary traumatic stress in more specific nursing populations, particularly in emergency, intensive care, oncology, and palliative care settings, and should use longitudinal designs to better clarify the relationships among perceived stress, burnout, secondary traumatic stress, and quality of life.

Study limitations

The results of this study should be interpreted in light of several limitations. First, the study was cross-sectional in design, which means that temporal ordering between variables could not be established and causal conclusions cannot be drawn. Accordingly, the mediation model tested in this study should be interpreted as statistical and associative rather than causal. In addition, the sample was predominantly female (84%), which reflects the gender structure of the nursing profession but may limit the generalizability of the findings to male nurses. Although the mediation model is theoretically justified, the relationships between variables should be further verified by longitudinal studies. Furthermore, the data were collected using the self-report method, which opens up the possibility of response bias and the influence of socially desirable responding. The sample is convenient and limited to one healthcare institution, so the generalizability of the findings to other nurse populations is limited. Also, only the physical and psychological domains of the WHOQOL-BREF were included in the model. Although these domains were considered most directly related to the study aims, this choice limits the comprehensiveness of the findings, as the social and environmental domains may also be relevant for understanding nurses’ well-being, particularly in relation to social support, workplace conditions, and broader contextual factors. Future research should therefore include all domains of quality of life in order to provide a more comprehensive understanding of nurses’ professional well-being.

## 5. Conclusions

The results of this study showed that perceived stress has a significant negative direct effect on the physical and psychological dimensions of nurses’ quality of life, while at the same time it has a significant positive effect on burnout and secondary traumatic stress. Burnout was shown to be a significant mediator in the relationship between perceived stress and both examined dimensions of quality of life, which indicates that burnout represents an important psychological mechanism through which stress contributes to the deterioration of nurses’ well-being. On the other hand, the mediating role of secondary traumatic stress was not confirmed, although this construct showed a specific connection with the psychological dimension of quality of life.

The obtained findings confirm the importance of professional stress and burnout in understanding nurses’ quality of life and indicate the need for systematic measures aimed at preserving their psychological and physical well-being. In practical terms, the results emphasize the importance of early recognition of professional stress, prevention of burnout at work, and development of organizational and individual interventions that can contribute to preserving the quality of life of nurses, and thus improving the quality and safety of healthcare.

## Figures and Tables

**Table 1:** Model fit indices for the structural equation model.

Index	Value
χ^2^	<0.001
df	0
*p*	— *
Comparative Fit Index (CFI)	1.000
Tucker–Lewis Index (TLI)	1.000
Normed Fit Index (NFI)	1.000
Incremental Fit Index (IFI)	1.000
Relative Noncentrality Index (RNI)	1.000
Standardized Root Mean Square Residual (SRMR)	<0.001
Goodness of Fit Index (GFI)	1.000
McDonald Fit Index (MFI)	1.000
Expected Cross Validation Index (ECVI)	0.095
Akaike Information Criterion (AIC)	3201
Bayesian Information Criterion (BIC)	3253
Sample-Size Adjusted BIC (SSABIC)	3208

Note: χ^2^—chi-square test of the model; df—degrees of freedom; *p*—statistical significance; * The *p*-value is not presented because the model was saturated (df = 0), and therefore the chi-square test of model fit was not interpretable.

**Table 2:** Descriptive statistics for study variables.

	Min	Max	M ± SD
Physical quality of life	39.29	100	75.32 ± 12.86
Psychological quality of life	29.17	100	74.76 ± 13.58
Perceived stress	13	25	19.21 ± 2.07
Burnout	10	48	32.40 ± 5.61
Secondary traumatic stress	11	48	29.69 ± 6.00

Note: Min—minimum value; Max—Maximum value; M—arithmetic mean; SD—standard deviation.

**Table 3:** Pearson correlations between quality of life dimensions, perceived stress, burnout, and secondary traumatic stress.

		1	2	3	4	5
1. Physical quality of life	r	—				
	*p*					
2. Psychological quality of life	r	0.160	—			
	*p*	0.006				
3. Perceived stress	r	−0.362	−0.304	—		
	*p*	<0.001	<0.001			
4. Burnout	r	−0.351	−0.359	0.230	—	
	*p*	<0.001	<0.001	<0.001		
5. Secondary traumatic stress	r	−0.129	−0.188	0.171	0.137	—
	*p*	0.027	0.001	0.003	0.018	

Note: r—Pearson correlation coefficient; *p*—statistical significance.

**Table 4:** Explained variance of mediators and outcome variables in the structural model.

	R^2^
Burnout	0.053
Secondary traumatic stress	0.029
Physical quality of life	0.208
Psychological quality of life	0.193

Note: R^2^—coefficient of determination.

**Table 5:** Standardized regression coefficients in the structural model.

Outcome	Predictor	β	SE	z	*p*	95% CI
Secondary traumatic stress	Perceived stress	0.171	0.055	3.120	0.002	0.064 to 0.278
Physical quality of life	Perceived stress	−0.291	0.048	−6.098	<0.001	−0.385 to −0.198
Physical quality of life	Burnout	−0.278	0.050	−5.569	<0.001	−0.376 to −0.180
Physical quality of life	Secondary traumatic stress	−0.041	0.056	−0.735	0.463	−0.150 to 0.068
Psychological quality of life	Perceived stress	−0.217	0.054	−4.008	<0.001	−0.324 to −0.111
Psychological quality of life	Burnout	−0.294	0.051	−5.769	<0.001	−0.394 to −0.194
Psychological quality of life	Secondary traumatic stress	−0.111	0.053	−2.100	0.036	−0.214 to −0.007
Burnout	Perceived stress	0.230	0.055	4.205	<0.001	0.123 to 0.337

Note: β—standardized regression coefficient; SE—standard error; *p*—statistical significance; CI—confidence interval.

**Table 6:** Indirect and total effects in the mediation model.

Effect	β	SE	z	*p*	95% CI
Indirect effect of perceived stress on physical quality of life through burnout	−0.064	0.019	−3.362	<0.001	−0.101 to −0.027
Indirect effect of perceived stress on physical quality of life through secondary traumatic stress	−0.007	0.010	−0.703	0.482	−0.027 to 0.013
Total indirect effect on physical quality of life	−0.071	0.022	−3.262	0.001	−0.114 to −0.028
Total effect on physical quality of life	−0.362	0.045	−8.035	<0.001	−0.450 to −0.274
Indirect effect of perceived stress on psychological quality of life through burnout	−0.068	0.020	−3.312	<0.001	−0.108 to −0.028
Indirect effect of perceived stress on psychological quality of life through secondary traumatic stress	−0.019	0.011	−1.797	0.072	−0.040 to 0.002
Total indirect effect on psychological quality of life	−0.087	0.023	−3.782	<0.001	−0.132 to −0.042
Total effect on psychological quality of life	−0.304	0.051	−5.944	<0.001	−0.404 to −0.204

Note: β—standardized effect coefficient; SE—standard error; *p*—statistical significance; CI—confidence interval.

## Data Availability

The data presented in this study are available on request from the first or corresponding author.
